# Diversity Trends in the United States Surgical Society Leadership From 1980 to 2025

**DOI:** 10.7759/cureus.101311

**Published:** 2026-01-11

**Authors:** Ashwin Govindan, Ari Ettleson, Justin M Robbins, Peter Ekeh, Anil Hingorani

**Affiliations:** 1 Medical Sciences, Quinnipiac University, Frank H. Netter MD School of Medicine, North Haven, USA; 2 Medicinal Sciences, George Washington University School of Medicine and Health Sciences, Washington, DC, USA; 3 Surgery, Boonshoft School of Medicine, Wright State University, Dayton, USA; 4 Surgery, New York University-Langone Health, New York, USA

**Keywords:** healthcare leadership, inclusion and diversity, retrospective research, sociodemographic, surgery general

## Abstract

Background

Physicians from underrepresented groups in medicine have historically been less likely to enter surgical specialties. This project sought to evaluate changes in the demographics of surgical society leadership from 1980 to 2025.

Methodology

In total, 31 societies were included in this retrospective analysis, and data on age, sex, and ethnicity were gathered and analyzed using SPSS.

Results

There was a significant increase in the number of female presidents and non-White presidents in the 2010-2025 period compared to 1980-1995. The Association for Academic Surgery (AAS), American Society of Breast Surgeons, and the Association for Surgical Education all had more female presidents than average, while the American Urological Association had fewer. AAS and the Surgical Society of the Alimentary Tract both had more non-White presidents than other societies. The American Pediatric Surgical Association and Western Surgical Association presidents were significantly older, while the AAS, American Society of Colon and Rectal Surgeons, EAST Trauma Society, Midwestern Surgical Society, and Society for University Surgeons presidents were significantly younger than the overall median age.

Conclusions

Many surgical societies have improved their representation of women and non-White surgeons. These significant changes in representation signify an appreciation for the demographic changes in the United States within the past 45 years.

## Introduction

The discourse surrounding diversity and inclusion in medicine has grown over the past two decades, leading to a focus on increasing representation in surgical and medical leadership [1,2]. This emphasis is grounded in the recognition of the advantages provided by diverse healthcare teams, which include the mitigation of healthcare disparities among patients from underrepresented backgrounds, increased patient trust and satisfaction, and improved treatment adherence [3,4]. A diverse physician workforce also promotes collaboration within healthcare teams [5-7].

Ensuring the physician workforce is diverse is critical in addressing healthcare disparities. Groups that are underrepresented in medicine (URiM) are defined as American Indian/Alaska Native, Black/African American, or Latino/Hispanic. Physicians who identify as URiM are more likely to work in primary care specialties and are more likely to practice in regions that lack adequate access to healthcare [7,8]. As the United States population diversifies, the physician workforce is falling behind, particularly in surgical specialties [9,10].

Surgical societies play a vital role in developing and implementing the policies that promote diverse workforces. Leaders of surgical societies wield considerable influence with regard to allocating resources, implementing policies, and charting the trajectory of the field [11]. Societies serve as platforms for professional development, networking, and ongoing education, training, and mentorship. Ensuring that society leadership is diverse, and not just its membership, is paramount.

Relative to other medical fields, graduating URiM students are less likely to enter general surgery or surgical subspecialty programs [12]. The lack of diversity in academic surgical departments has been well documented, and recent studies have shown that, although diversity in surgical society leadership has increased, non-White females are heavily underrepresented [13-15]. Societies that were the first to diversify their leadership by gender (i.e., female, White president) were more likely to have racially diverse leadership sooner [16].

Within discussions of diversity and inclusion in professional settings, the metaphors of the “glass ceiling” and “sticky floors” have gained prominence. The “glass ceiling” refers to invisible barriers that prevent women and URiMs from advancing to senior positions, while “sticky floors” represent the phenomenon of being relegated to lower-level positions with limited opportunities for advancement [17]. These metaphors highlight the systemic barriers to diverse leadership.

As a field striving for inclusive, representative leadership, setting realistic goals necessitates a thorough assessment of the present. To chart a course forward, it is imperative to understand the current position. Comprehensive data, however, on the demographics of surgical society leadership remains scarce. This study aims to investigate trends in age, perceived gender, and perceived race in the presidents of surgical societies in the United States from 1980 through 2025.

## Materials and methods

This was a retrospective study evaluating the demographics of presidents of surgical societies from 1980 to 2025. This time period was chosen to provide enough historical depth to meaningfully observe change while minimizing incomplete data. Surgical societies that were included comprised flagship national societies, subspecialty societies, and regional societies. The inclusion criteria for any given society were that they represented general or subspecialty surgery, maintained publicly available data dating to at least the early 1980s, and had a clearly defined presidential role. President-elects for the 2025-2026 term were included to account for all societies that had elections in 2025 and had either a president who had begun their tenure or were soon to start. Both regional and national societies were included to ensure as broad a geographic scope as possible. In total, 31 societies were included, encompassing societies with large membership bases, visibility in academia, and sustained activity.

The societies are as follows: American Academy of Orthopedic Surgeons (AAOS), American Academy of Otolaryngology (AAO), American Association of Endocrine Surgeons (AAES), American Association for the Surgery of Trauma (AAST), American Congress of Obstetricians and Gynecologists (ACOG), American College of Surgeons (ACS), American Pediatric Surgical Association (APSA), American Surgical Association (ASA), American Society of Breast Surgeons (ASBS), American Society of Colon and Rectal Surgeons (ASCR), American Society for Metabolic and Bariatric Surgery (ASMBS), American Society of Plastic Surgeons (ASPS), American Society of Transplant Surgeons (ASTS), American Urological Association (AUA), Association for Academic Surgery (AAS), Association for Surgical Education (ASE), Central Surgical Association (CSA), EAST Trauma Society (EAST), Midwestern Surgical Society (MSS), New England Surgical Society (NESS), North Pacific Surgical Society (NPSS), Society of American Gastrointestinal and Endoscopic Surgeons (SAGES), Society for Surgery of the Alimentary Tract (SSAT), Society of Surgical Oncology (SSO), Society of Thoracic Surgery (STS), Southern Surgical Association (SSA), Society for University Surgeons (SUS), Society of Vascular Surgery (SVS), Southwestern Surgical Society (SWSS), Western Surgical Association (WSA), and Western Trauma (WT).

Age, race, and visible gender/sex were determined via institutional profiles, online obituaries, and HealthGrades. The year of birth and the year of presidency were used to calculate age at the time of presidency. Age was used instead of stage of practice due to significantly more availability of complete data. Race was classified into White and non-White using the United States Census classifications. An inter-rater agreement percentage of 80% was required for the classification to be accepted and standardized. Through review of publicly available images, biographies, institutional sources, and inter-rater agreement percentages, a composite “perceived race” was composed. If there was evidence of multiple ethnicities, the individual was included within the category that was most visually perceived and with the highest degree of inter-rater agreement. Visible sex was binary (i.e., male or female) and determined by the rater using available pronouns, institutional descriptions, or other publicly available information to create a composite “perceived gender,” with a required agreement percentage of 80% between raters.

All 31 societies were contacted by study coordinators using a templated message asking for basic demographic data on their presidents since 1980. Of the 31 societies contacted, none provided this information. If any one of the demographic categories was unable to be found via online search, it was registered as a missing value and excluded from analysis. In the event of a discrepancy between sources (i.e., institutional vs. obituary), institutional information was prioritized. Four presidents could not be identified using these methods, each of whom was the most recent president of their societies. Societies that were founded after 1980 are as follows: ASBS, ASE, ASMBS, EAST, NPSA, and SAGES. As such, fewer of their presidents were included. There were 196 presidents for whom age could not be identified. One society, the Association of Program Directors in Surgery, lacked any publicly available information about its presidents and was therefore removed from this study.

Statistical analysis was performed using SPSS version 29.0 (IBM Corp., Armonk, NY, USA). Descriptive and frequency analyses were used for demographic data. Chi-square, Kruskal-Wallis, and a two-tailed independent-samples t-test were used for significance testing. Chi-square analyses were conducted as omnibus tests, and standardized residuals were used to descriptively characterize societies meaningfully deviating from the average. The primary analyses comprised hypothesis-driven comparisons (i.e., time period and gender, time period and race, and time period and median age), and adjustments for multiple comparisons were not applied nor deemed necessary. Demographic variables were compared from the earliest time period to the latest time period (1980-1995 and 2010-2025, respectively), with a middle period omitted to maximize contrast between the earliest and most recent eras and to determine significant changes in demographic representation. A p-value less than 0.05 was considered statistically significant.

## Results

A total of 1,360 presidents from 31 surgical societies were included in the analysis. A breakdown of age, perceived race, and perceived gender (binary) delineated by the surgical society can be found in Table 1. From 1980 to 2025, the median age of surgical society presidents was 58.0 (n = 1,164, IQR = 11), and the mean age of surgical society presidents was 57.6 (n included = 1,164, n missing = 196, SD = 8.4) (Table 1).

**Table 1:** Demographics of surgical societies. ACS = American College of Surgeons; AAS =  Association for Academic Surgery; AAOS = American Academy of Orthopedic Surgeons; AAO = American Academy of Otolaryngology; AAES = American Association of Endocrine Surgeons; AAST = American Association for the Surgery of Trauma; ACOG = American Congress of Obstetricians and Gynecologists; APSA = American Pediatric Surgical Association; ASMBS = American Society for Metabolic and Bariatric Surgery; ASA = American Surgical Association; ASBS = American Society of Breast Surgeons; ASCRS = American Society of Colon and Rectal Surgeons; ASPS = American Society of Plastic Surgeons; ASTS = American Society of Transplant Surgeons; AUA = American Urological Association; ASE = Association for Surgical Education; CSA = Central Surgical Association; EAST = EAST Trauma Society; MSS = Midwestern Surgical Society; NESS = New England Surgical Society; NPSS = North Pacific Surgical Society; SAGES = Society of American Gastrointestinal and Endoscopic Surgeons; SSA = Southern Surgical Association; SSAT = Society for Surgery of the Alimentary Tract; SSO = Society of Surgical Oncology; STS = Society of Thoracic Surgery; SUS = Society for University Surgeons; SVS = Society of Vascular Surgery; SWSC = Southwestern Surgical Society; WSA = Western Surgical Association; WT = Western Trauma

Society	Age (mean)	SD	Age (median)	IQR	Race (non-White)	Sex (female)	Years included in the analysis
AAES	57.36	10.40	56.00	15	6 (13.0%)	5 (10.9%)	1980–2025
AAO	57.79	6.50	59.00	9	6 (13.0%)	5 (10.9%)	1980–2025
AAOS	57.90	5.48	58.00	6	1 (2.2%)	1 (2.2%)	1980–2025
AAS	44.21	2.41	44.00	4	12 (26.1%)	9 (19.6%)	1980–2025
AAST	57.11	8.31	59.00	10	2 (4.3%)	4 (8.7%)	1980–2025
ACOG	60.63	6.18	62.00	9	3 (6.7%)	8 (17.8%)	1980–2024
ACS	67.23	4.78	66.50	7	4 (8.7%)	6 (13.0%)	1980–2025
APSA	63.33	6.58	62.00	6	3 (6.5%)	6 (13.0%)	1980–2025
ASA	64.47	5.05	64.00	8	5 (11.4%)	3 (6.8%)	1980–2024
ASBS	52.15	9.71	51.50	17	2 (6.7%)	11 (36.7%)	1996–2025
ASCRS	57.72	5.55	56.00	6	3 (6.5%)	5 (10.9%)	1980–2025
ASE	49.33	5.88	50.00	7	2 (4.4%)	9 (20.0%)	1981–2025
ASMBS	57.94	9.18	58.00	15	4 (9.5%)	4 (9.5%)	1983–2024
ASPS	56.14	4.40	56.00	5	3 (6.5%)	3 (6.5%)	1980–2025
ASTS	50.73	8.83	51.50	14	4 (8.7%)	8 (17.4%)	1980–2025
AUA	66.62	5.16	66.00	9	6 (12.8%)	0	1980–2025
CSA	58.24	6.14	59.00	8	1 (2.2%)	2 (4.3%)	1980–1999, 2001–2025
EAST	48.00	4.21	48.00	4	2 (5.1%)	5 (12.8%)	1987–2025
MSS	54.83	6.19	54.00	5	1 (2.2%)	3 (6.5%)	1980–2025
NESS	64.65	5.93	64.00	8	0	2 (4.4%)	1980–2024
NPSA	56.47	5.72	58.00	9	4 (15.4%)	1 (3.8%)	2000–2025
SAGES	52.00	6.00	51.00	8	2 (4.5%)	4 (9.1%)	1981–2024
SSA	63.31	7.29	63.00	7	3 (6.5%)	1 (2.2%)	1980–2025
SSAT	58.56	5.08	59.00	8	7 (20.0%)	3 (8.6%)	1990–2024
SSO	57.00	5.18	57.00	6	1 (2.2%)	5 (10.9%)	1980–2025
STS	60.97	6.77	61.00	7	2 (4.4%)	1 (2.2%)	1980–2025
SUS	46.30	4.12	45.00	4	6 (13.0%)	7 (15.2%)	1980–2025
SVS	61.23	5.54	61.00	8	4 (8.7%)	1 (2.2%)	1980–2025
SWSC	56.57	5.56	56.00	10	7 (15.6%)	4 (8.9%)	1980–2025
WSA	60.85	6.15	61.00	7	2 (4.3%)	5 (10.9%)	1980–2025
WT	55.11	7.11	56.50	9	0	4 (8.7%)	1980–2025

**Figure 1:**
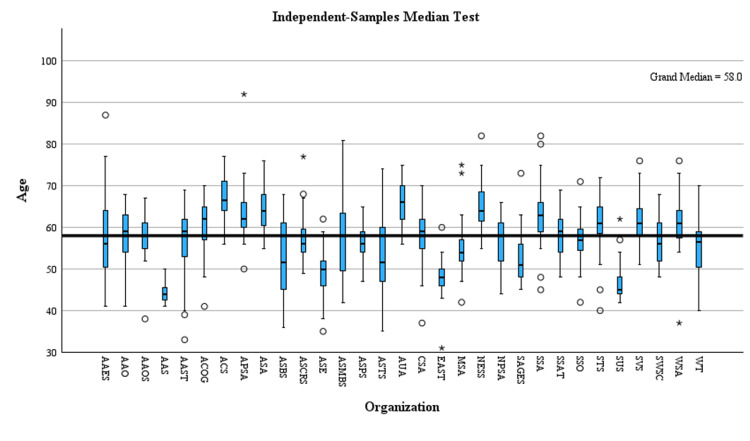
Median age by organization. Circle = outliers; asterisk = significant finding. ACS = American College of Surgeons; AAS =  Association for Academic Surgery; AAOS = American Academy of Orthopedic Surgeons; AAO = American Academy of Otolaryngology; AAES = American Association of Endocrine Surgeons; AAST = American Association for the Surgery of Trauma; ACOG = American Congress of Obstetricians and Gynecologists; APSA = American Pediatric Surgical Association; ASMBS = American Society for Metabolic and Bariatric Surgery; ASA = American Surgical Association; ASBS = American Society of Breast Surgeons; ASCRS = American Society of Colon and Rectal Surgeons; ASPS = American Society of Plastic Surgeons; ASTS = American Society of Transplant Surgeons; AUA = American Urological Association; ASE = Association for Surgical Education; CSA = Central Surgical Association; EAST = EAST Trauma Society; MSS = Midwestern Surgical Society; NESS = New England Surgical Society; NPSS = North Pacific Surgical Society; SAGES = Society of American Gastrointestinal and Endoscopic Surgeons; SSA = Southern Surgical Association; SSAT = Society for Surgery of the Alimentary Tract; SSO = Society of Surgical Oncology; STS = Society of Thoracic Surgery; SUS = Society for University Surgeons; SVS = Society of Vascular Surgery; SWSC = Southwestern Surgical Society; WSA = Western Surgical Association; WT = Western Trauma

APSA and WSA had a median age of presidents significantly older than the median age, while AAS, ASCRS, EAST, MSA, and SUS had a median age of presidents significantly under the median age (Figure 1).

A two-tailed independent-samples t-test was used to determine how the mean age for a surgical society president has changed from the earliest to the latest 15-year time period. Equal variances were not assumed (Levene’s test for equality of variances, p = 0.004), and significant differences were determined between the 1980-1995 group and the 2010-2025 group (t = -4.3, df = 747.9, p < 0.001). The mean age was determined to be significantly younger in the 1980-1995 group (n = 375, mean = 56.0, SD = 8.7) than in the 2010-2025 group (n = 432, mean = 58.9, SD = 7.6).

**Figure 2:**
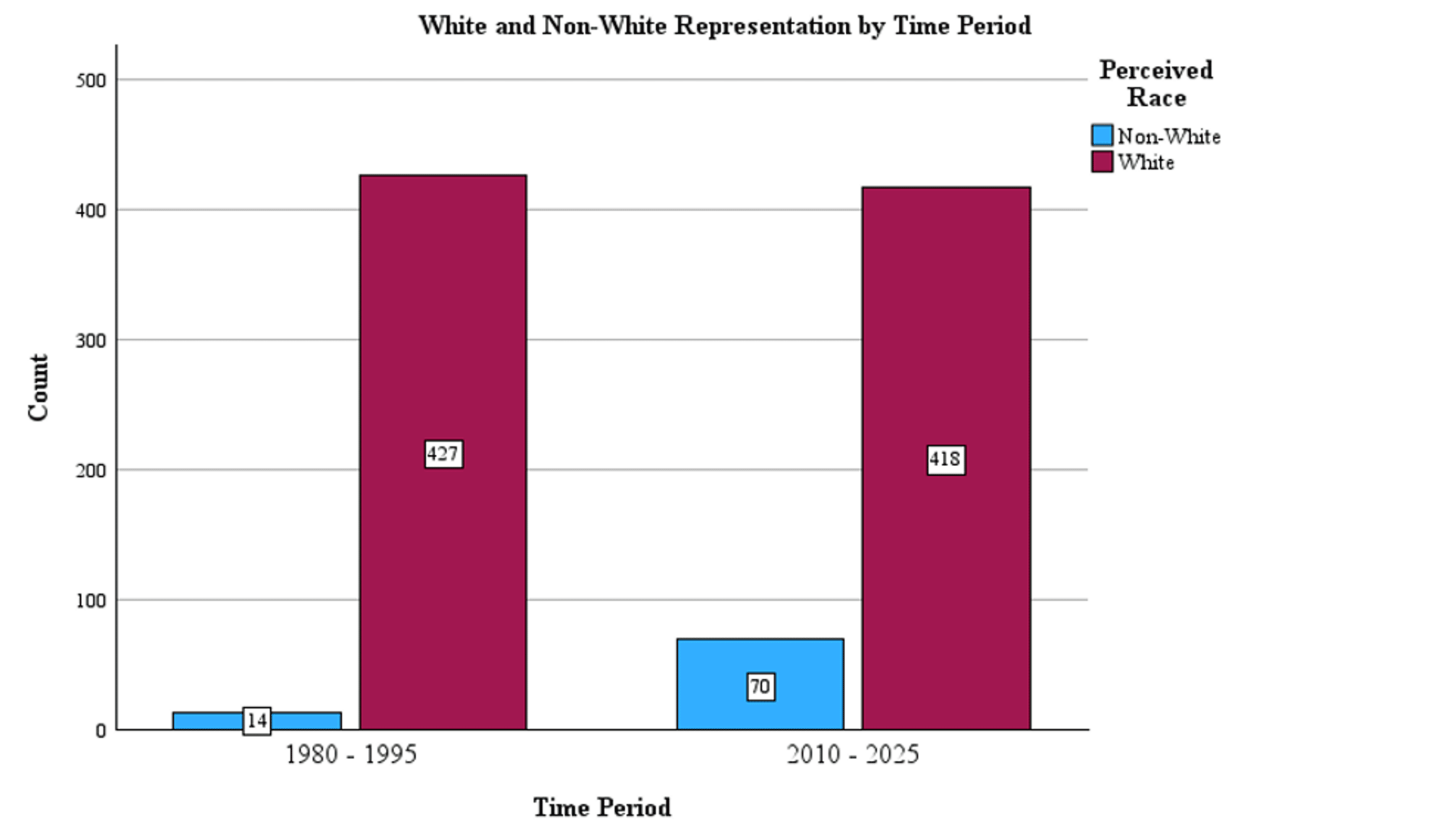
Changes in White vs. non-White representation by time period.

Race (White or non-White) was determined for all 1,360 presidents. Overall, 7.9% (n = 108) of presidents were non-White, and 92.1% (n = 1,252) were White. There were significant increases (chi-square = 35.1, df = 1, p < 0.001) in the number of non-White presidents from 14 to 70 from the earliest period (1980-1995) to the latest period (2010-2025), respectively (Figure 2, Table 2).

**Table 2:** Chi-square significance testing.

Test	Chi-square value	Degrees of freedom	P-value
Time period x gender	80.85	1	0.001
Time period x race	35.14	1	0.001
Organization x gender	66.51	30	0.001
Organization x race	61.73	30	0.001

**Figure 3:**
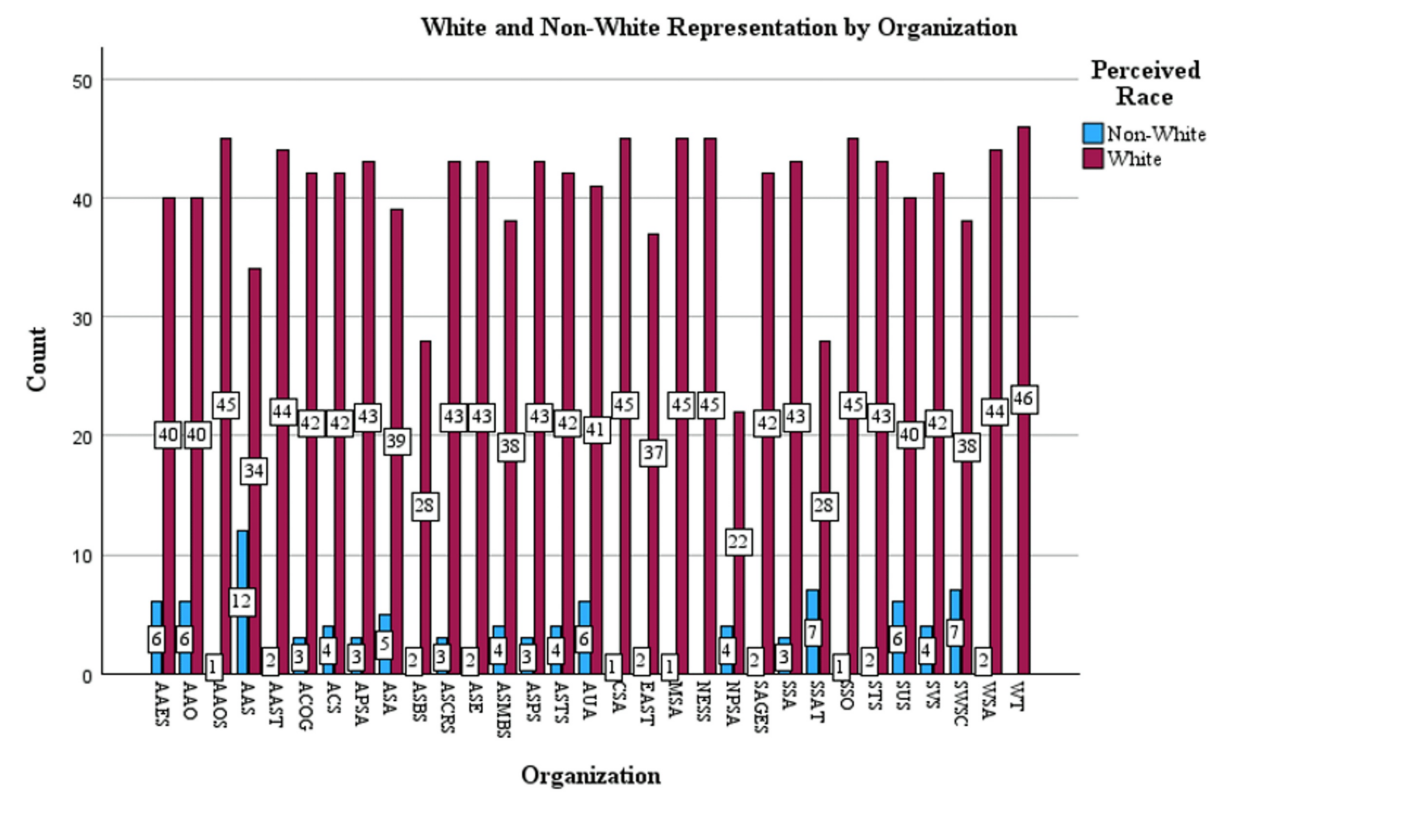
White vs. non-White representation by organization. ACS = American College of Surgeons; AAS =  Association for Academic Surgery; AAOS = American Academy of Orthopedic Surgeons; AAO = American Academy of Otolaryngology; AAES = American Association of Endocrine Surgeons; AAST = American Association for the Surgery of Trauma; ACOG = American Congress of Obstetricians and Gynecologists; APSA = American Pediatric Surgical Association; ASMBS = American Society for Metabolic and Bariatric Surgery; ASA = American Surgical Association; ASBS = American Society of Breast Surgeons; ASCRS = American Society of Colon and Rectal Surgeons; ASPS = American Society of Plastic Surgeons; ASTS = American Society of Transplant Surgeons; AUA = American Urological Association; ASE = Association for Surgical Education; CSA = Central Surgical Association; EAST = EAST Trauma Society; MSS = Midwestern Surgical Society; NESS = New England Surgical Society; NPSS = North Pacific Surgical Society; SAGES = Society of American Gastrointestinal and Endoscopic Surgeons; SSA = Southern Surgical Association; SSAT = Society for Surgery of the Alimentary Tract; SSO = Society of Surgical Oncology; STS = Society of Thoracic Surgery; SUS = Society for University Surgeons; SVS = Society of Vascular Surgery; SWSC = Southwestern Surgical Society; WSA = Western Surgical Association; WT = Western Trauma

AAS and SSAT both had more non-White presidents than other societies between 1980 and 2025, as indicated by a standardized residual value greater than 2, indicating that they were outliers in the number of non-White presidents in their societies (Figure 3).

**Figure 4:**
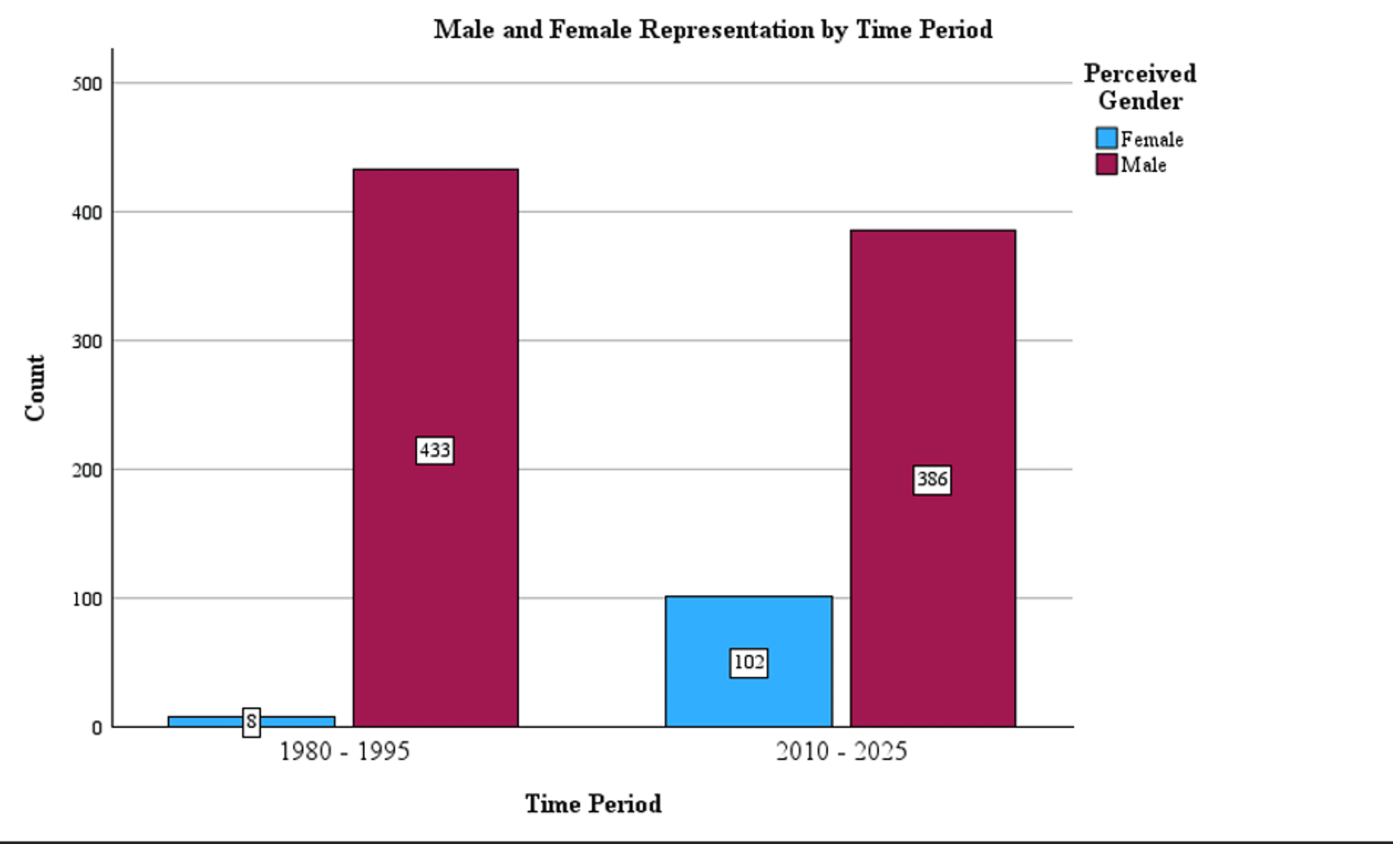
Changes in male vs. female representation by time period.

Perceived gender was included for all presidents. Overall, 11.8% (n = 110) of presidents were female, and 88.2% (n = 1250) were male. There were significant increases (chi-square = 80.8, df = 1, p < 0.001) in the number of women who were surgical society presidents from 2010-2025 (n = 102) compared to 1980-1995 (n = 8) (Figure 4, Table 2).

**Figure 5:**
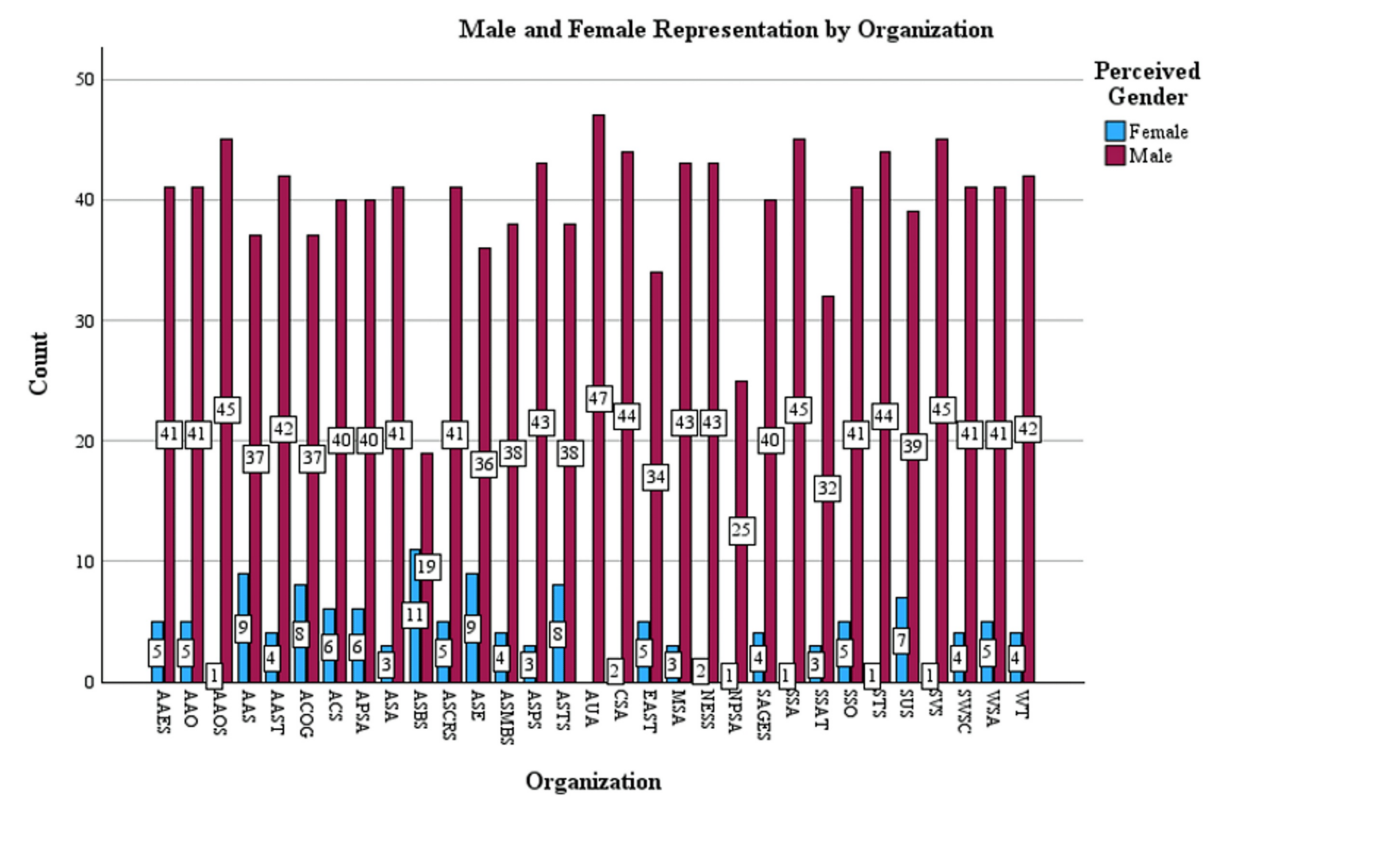
Male vs. female representation by society. ACS = American College of Surgeons; AAS =  Association for Academic Surgery; AAOS = American Academy of Orthopedic Surgeons; AAO = American Academy of Otolaryngology; AAES = American Association of Endocrine Surgeons; AAST = American Association for the Surgery of Trauma; ACOG = American Congress of Obstetricians and Gynecologists; APSA = American Pediatric Surgical Association; ASMBS = American Society for Metabolic and Bariatric Surgery; ASA = American Surgical Association; ASBS = American Society of Breast Surgeons; ASCRS = American Society of Colon and Rectal Surgeons; ASPS = American Society of Plastic Surgeons; ASTS = American Society of Transplant Surgeons; AUA = American Urological Association; ASE = Association for Surgical Education; CSA = Central Surgical Association; EAST = EAST Trauma Society; MSS = Midwestern Surgical Society; NESS = New England Surgical Society; NPSS = North Pacific Surgical Society; SAGES = Society of American Gastrointestinal and Endoscopic Surgeons; SSA = Southern Surgical Association; SSAT = Society for Surgery of the Alimentary Tract; SSO = Society of Surgical Oncology; STS = Society of Thoracic Surgery; SUS = Society for University Surgeons; SVS = Society of Vascular Surgery; SWSC = Southwestern Surgical Society; WSA = Western Surgical Association; WT = Western Trauma

AAS, ASBS, and ASE all had standardized residual values greater than 2, indicating that they were outliers in the number of female presidents in their societies and had meaningfully more female presidents than the average. AUA had fewer female presidents than expected, with a standardized residual of less than 2 (Figure 5).

## Discussion

A diverse workforce positively impacts clinical outcomes and patient care. This study explores the current state of diversity in surgical society leadership by analyzing the age, sex/visible gender, and race/ethnicity of presidents from major general surgery and surgical subspecialty societies since 1980. While there were significant increases in the representation of women and non-White individuals since 1980, true progress for URiM groups is not supported by the results of this study and requires a more granular study. It is important to acknowledge the discrepancy between URiM and non-White insofar as non-White is broadly defined and includes groups that are not historically underrepresented in medicine (e.g., Asian Americans), and our findings may suggest an overestimation of improvements in representation in presidents from URiM backgrounds.

This analysis suggests that various societies are diversifying their leadership. The number of female presidents has increased from 8 in the earliest period (1980-1995) to 102 in the most recent time period (2010-2025). AAS, ASBS, and ASE all had more female presidents than other societies, signifying trends in female-oriented specialties and academic medicine. AAS and ASE are both academic societies and have likely benefited from diversity initiatives that have become more commonplace in universities and their affiliated hospital systems, focusing on elevating female surgeons who have become more numerous in recent decades. ASBS has a clinical focus on breast surgery, and it is a natural consequence of this society’s scope of practice to have significantly more female representation than other societies. Conversely, AUA, focused on urological health, had fewer female presidents than was expected, indicating that not all surgical societies are diversifying their leadership and may be reflective of lower numbers of women entering fields such as urology, which have long been male-dominated. There were also notable increases in racial representation from the earliest (14 non-White presidents) to the most recent time period (70 non-White presidents). AAS and SSAT both had more non-White presidents than was expected, indicating, as with gender, a trend towards increasing representation in academic medicine.

Although there were statistically significant differences in the median age of surgical society presidents in the earliest period vs. the latest (56 and 58, respectively), the relative proximity of these ages suggests that the age of leadership has remained largely stable. Potential factors for presidents being older in the more recent period include longer career paths to leadership, academic productivity peaking at later stages, or a more modern emphasis on increased time in practice for leadership. Society size was not considered a confounding variable within the analysis, as the number of presidents was not dependent upon society size. It is important to acknowledge that the demographics of each society are dependent upon the nature of the society (e.g., academic vs. regional), which may be, in part, responsible for some of the observed demographic changes.

The benefits of diversity apply at all levels of medical education, not just at executive levels. Medical students who attend more diverse programs are more prepared and motivated to care for diverse populations in residency [18]. Similar trends are observed at the residency level, where programs that emphasize diversity in their training tend to produce residents who are better equipped to address the healthcare needs of diverse patient populations [19]. Healthcare workforces with team members from different backgrounds improve patient outcomes [20]. In a country with increasing diversity, notably with large shifts in the past half-century in the make-up of United States physicians (particularly in Asian immigrants), emphasizing diversity from the earliest stages of medical training will not only benefit physicians but also the patients for whom they care [21].

The findings of this study suggest there have been significant improvements in the last decade to appropriately capture the more diverse physician workforce, and many societies have shown a commitment to this issue. The formation of Diversity, Equity, and Inclusion committees and initiatives has become increasingly common among medical and surgical societies to advance surgeons of URiM backgrounds through targeted outreach and mentorship initiatives. For example, SVS offers a scholarship that awards medical and pre-medical students from URiM backgrounds the opportunity to attend their annual conference expense-free. AUA has shown commitment to diversifying its leadership by establishing the Leadership in Education, Achievement, and Diversity Program to develop URiM leaders in urologic research and offers urology residents funding, mentorship, and networking [22].

Limitations

Throughout the data collection process, it was difficult to obtain demographic information (particularly age) about the various past presidents. Some data points were excluded from this analysis because society websites did not list presidents for particular years, and the ages of some presidents could not be found. Although information was not captured from the societies themselves, information was still gathered from official society websites. While this was the only possibility for this project due to the lack of response to study coordinators, it may have introduced confounds into the data. It is also necessary to recognize that this is not a comprehensive analysis of all surgical societies, nor does it capture all levels of leadership, many of which may have also changed in the included time frame. The decision to focus solely on presidents was made to ensure as complete a dataset as possible, but ultimately resulted in the omission of possible trends for other positions, such as executive committees.

This study was limited by the number of demographic characteristics that were included. Age, sex/visible gender, and race/ethnicity may be defining characteristics, but do not perfectly encapsulate an individual. As previously stated, the use of perceived demographics introduced a layer of methodological uncertainty that could be limited in future studies. Similarly, non-White and URiM are not synonymous, and this study only explicitly investigates changes in non-White representation. Given the retrospective nature and the methods of data collection, other forms of diversity (e.g., socioeconomic status, urban vs. rural, sexual orientation, or IMG vs. US trainees), which were not publicly available, had to be excluded.

## Conclusions

Diversity among surgical society presidents has increased significantly since 1980. There are observable trends of increased representation of non-White presidents and female presidents in the last 15 years. These changes may be attributed to surgical society initiatives or a growing appreciation of a diverse, changing nation. A commitment to diversity in medicine allows for a physician workforce to evolve so it may more accurately represent its patient population. Future research could evaluate changes in the representation of executive council and committee members, groups that hold considerable sway over the policy of surgical societies, who could, in the future, formally report URiM-specific leadership data, encourage pipeline programs, and prioritize diversity at all levels of leadership. In light of recent shifts in federal policy toward minority groups and immigrants, particularly regarding civil liberties, higher-education, and healthcare access, maintaining a thorough understanding of the changing demographics of the United States so there may be an appropriate reflection in the leadership of medical societies remains, perhaps, as vital as ever.
